# PAHs-induced metabolic aberrations and intact circadian rhythms in zebrafish: a promising approach for aquatic surveillance

**DOI:** 10.1038/s41598-025-15368-z

**Published:** 2025-08-19

**Authors:** Meng Chen, Senmei He, Zongming Ren

**Affiliations:** College of Geography and Environment, Shandong Normal University, Jinan, 250358 P. R. China

**Keywords:** Metabolism, Phenanthrene, Pyrene, Real-time biomonitoring, Ecology, Ecology, Environmental sciences

## Abstract

Polycyclic aromatic hydrocarbons (PAHs), recognized as teratogenic and carcinogenic pollutants, exhibit moderate-to-high acute toxicity and long-term detrimental effects on aquatic organisms. However, conventional monitoring methods often fail to detect early-stage sublethal impacts of PAHs, potentially due to limited disruption of intrinsic circadian rhythms during initial exposure periods. To investigate the physiological responses of aquatic species to PAHs, we employed real-time biomonitoring to evaluate metabolic alterations in juvenile zebrafish (*Danio rerio*), including oxygen consumption rate (OR), carbon dioxide excretion rate (CR), ammonia-nitrogen excretion rate (AE), respiratory quotient (RQ), and ammonia quotient (AQ), alongside their circadian rhythmicity under PAH exposure. Two representative PAHs, phenanthrene (Phe) and pyrene (Pyr), were tested at 10%×LC_50_(96 h) concentrations (Phe: 102 µg/mL; Pyr: 70.6 µg/mL) over a 7-day exposure period. Comparative analysis revealed significant PAH-induced suppression of OR and CR, while AE was markedly enhanced, resulting in elevated RQ and AQ. Despite these metabolic responses, circadian rhythms remained intact across all parameters. These findings indicate that PAHs induce transient metabolic dysregulation without perturbing core circadian mechanisms, highlighting the feasibility of zebrafish metabolic profiling as a sensitive tool for aquatic PAH monitoring.

**Supplementary Information:**

The online version contains supplementary material available at 10.1038/s41598-025-15368-z.

## Introduction

The rapid progression of urbanization and industrialization has led to substantial waste influx into aquatic ecosystems through diverse pathways. Elevated contaminant loads in water bodies have precipitated severe environmental degradation, with myriad organic pollutants—intentionally or inadvertently discharged—posing significant ecotoxicological risks to ecological stability and human health, a pressing concern widely documented in multiple studies^1,2^. Key pollutant sources include: (1) agricultural inputs (e.g., pesticides)^3^; (2) anthropogenic mobilization of naturally occurring toxic geochemical agents, including heavy metals and metallic compounds^4^; and (3) petroleum-derived contaminants such as polycyclic aromatic hydrocarbons (PAHs) from fuel spills^5^. Among these, PAHs are notably hazardous to aquatic biota due to their moderate-to-high acute toxicity^6^.

Polycyclic aromatic hydrocarbons are characterized by two or more fused benzene rings, are classified as persistent organic pollutants (POPs) due to their environmental persistence and bioaccumulative potential^7^. Recognized for their teratogenic and carcinogenic properties, 16 PAHs have been designated, although arbitrarily, as priority pollutants by the U.S. Environmental Protection Agency (USEPA)^8^. These contaminants enter surface waters through diverse pathways, including agricultural pesticide runoff, plastic manufacturing effluents, chemical industrial discharges, domestic wastewater, and atmospheric deposition of particulate matter^9–11^. PAH contamination has been documented in rivers, lakes, and coastal systems across most nations, albeit with varying severity^12^. Notable examples include the detection of 20 PAH congeners in sediments from Nigeria’s Ikpoba River^13^, total PAH concentrations ranging from 277 to 4393 ng/L (mean: 1178 ng/L) in Laizhou Bay^14^, and PAH levels between 396.42 and 624.06 ng/L (mean: 436.99 ng/L) in surface waters of Jilin’s Dongliao River^15^.

The health impacts of polycyclic aromatic hydrocarbons (PAHs) on humans are primarily determined by the duration, pathways, and dose/concentration of exposure^16^. Acute toxicity from high-level PAH exposure in humans manifests as ocular irritation, nausea, vomiting, diarrhea, confusion, and dermal inflammation^17,18^. , while chronic exposure correlates with progressive health deterioration, including immunosuppression, cataract formation, renal/hepatic dysfunction, respiratory disorders (e.g., asthma and pulmonary abnormalities), erythema, and dermatitis^19^. PAHs exhibit significant bioaccumulation in fish and shellfish, with tissue concentrations substantially exceeding environmental levels^20^. Early developmental stages are particularly vulnerable, which show impaired swimming performance, growth retardation, skeletal deformities, and mortality following PAH exposure^21^. Cardiotoxicity and immunomodulatory dysfunction represent the most critical toxicological endpoints in fish^22^. Hepatic PAH accumulation has been linked to reduced chemotactic and phagocytic activities in aquatic species^23^. Experimental studies demonstrate suppressed serum lysozyme activity in rainbow trout (*Oncorhynchus mykiss*) following diesel exposure^24^, alongside diminished nonspecific cellular responses in medaka (*Oryzias latipes*) inhabiting PAH-contaminated waters^25^.

This study focuses on two representative PAHs, phenanthrene (Phe) and pyrene (Pyr). Phe, a three-ring PAH, is widely adopted as a model compound for PAH research and a biomarker for contamination monitoring due to its low molecular weight, high aqueous solubility, and bioavailability, which facilitate its uptake and toxicological impact in aquatic organisms^26,27^. Exposure to Phe induces alterations in antioxidant enzyme activity^28^, lipid peroxidation^29^, histopathological changes in aquatic species^30^, and acute lethality^31^. Owing to its lipophilic nature, Phe readily penetrates biological membranes and accumulates rapidly. Furthermore, its persistence and biomagnification potential through food chains pose direct threats to animals and humans, including hepatotoxicity, developmental toxicity, reproductive impairment, and genotoxicity. Pyr, a four-ring PAH, is another archetypal PAH with pronounced environmental accumulation. It is frequently employed as an indicator for PAH monitoring and a model molecule for studying PAH degradation^32^. Compared to low-molecular-weight PAHs, Pyr exhibits greater resistance to environmental removal^33^. Chronic exposure to Pyr at concentrations of 3–5 mg/m³ has been associated with systemic effects such as headaches, fatigue, sleep disturbances, appetite loss, elevated leukocyte counts, and increased erythrocyte sedimentation rates.

PAHs are characterized by low aqueous solubility, hydrophobicity, and lipophilicity in aquatic environments^34^, coupled with limited bioavailability^35^, which collectively complicate PAH their monitoring. Conventional water quality assessment methods rely on physicochemical parameters analyzed through techniques such as gas chromatography (GC)^36^, high-performance liquid chromatography (HPLC)^37^, capillary electrophoresis^38^, and surface-enhanced Raman spectroscopy (SERS)^39^. However, these approaches suffer from inherent limitations: they are technically complex, labor-intensive, and fail to provide timely warnings of contamination risks. Key drawbacks include (1) reliance on large volumes of organic solvents during extraction, potentially causing secondary environmental contamination; (2) dependency on costly instrumentation; (3) inability to perform real-time, on-site monitoring; (4) failure to assess ecological and human health risks posed by pollutants; and (5) time-lagged and discontinuous data acquisition.

To address the limitations of conventional monitoring methods, we propose the use of real-time biomonitoring technology based on biological indicators, which enables comprehensive environmental pollution assessment from a biological perspective^40^. Conventional biomonitoring encompasses two approaches: active and passive biomonitoring^41^. As an active biomonitoring method, real-time biomonitoring technology leverages organisms’ dynamic responses to environmental stressors, achieving real-time water quality evaluation through continuous tracking of biological parameters^42^. In metabolic studies, oxygen consumption rate (OR), carbon dioxide excretion rate (CR), ammonia-nitrogen excretion rate (AE), respiratory quotient (RQ), and ammonia quotient (AQ) are widely utilized biomarkers. Among these, OR exhibits the most direct correlation with metabolic activity in aquatic organisms^43,44^, while CR reflects metabolic rate variations^45^. To holistically assess stress responses, RQ was developed as a composite index integrating OR and CR, enabling both combined analysis of gas exchange and systemic metabolic evaluation^45,46^. AE, representing the terminal products of protein catabolism and amino acid deamination^47^, serves as a critical metric for quantifying metabolic effects of environmental stress in freshwater fish, which predominantly excrete nitrogen as ammonia^48^. AQ, modulated by both AE and OR, provides insights into protein composition and metabolic dynamics in fish^49^. Zebrafish are particularly suited for such investigations. As natural bioindicators for PAH contamination^50^, their high genomic homology with humans (87% similarity) positions them as ideal models for aquatic health risk assessment and metabolic research^51^.

The cumulative and latent toxicity of PAHs poses significant challenges for biological detection, as irreversible toxic effects often manifest before organisms exhibit discernible distress^52^. Given these considerations, this study explores the relationship between the absence of overt physiological disruptions exposure and the stability of intrinsic circadian rhythms, proposing the application of real-time biomonitoring technology to characterize the toxicological profiles of phenanthrene (Phe) and pyrene (Pyr) in aquatic organisms. Zebrafish were exposed to Phe (102 µg/mL) and Pyr (70.6 µg/mL) for 7 days, with continuous monitoring of metabolic parameters—OR, CR, AE, RQ, and AQ—alongside circadian rhythm analysis under PAH stress. We hypothesize that low-dose PAH toxicity in zebrafish can be characterized through metabolic perturbations. Utilizing real-time biomonitoring, this study aims to delineate chronic metabolic alterations induced by subacute Phe and Pyr exposure, thereby addressing the knowledge gap in PAH toxicity assessment. The findings are anticipated to establish a novel framework for environmental and health risk evaluation, advancing analytical methodologies for PAH toxicity profiling.

## Materials and methods

### Materials and reagents

Phenanthrene (≥ 95% purity, CAS:85-01-8) and pyrene (≥ 95% purity, CAS:129-00-0) were procured from Solarbio (Beijing, China). Dimethyl sulfoxide (DMSO, ≥ 95% purity, CAS:67-68-5), employed as a solvent for chemical preparation, was obtained from the China National Standard Sample Center (Beijing, China). The LH-N2N3-100 ammonia-nitrogen detection kit was supplied by Beijing Lianhua Yongxing Technology Development Co., Ltd. (Beijing, China).

Phe and Pyr exposure concentrations (102 µg/mL and 70.6 µg/mL, respectively) were standardized to 10%×LC_50_(96 h) values derived from prior zebrafish (Danio rerio) acute toxicity assays^53^. Stock solutions were prepared by dissolving Phe and Pyr in dimethyl sulfoxide (DMSO), with solvent concentrations maintained at < 0.5% (v/v)(The DMSO concentration (0.5% v/v) exceeds the OECD (2013) Test No. 236-recommended 0.01% threshold. Yet, studies (e.g., zebrafish early development assays) indicate 1% is safe, implying juveniles can tolerate 0.5%)^54^. Previous studies confirm that DMSO at this concentration exhibits no acute toxicity or locomotor interference in zebrafish^55^. All stock solutions were aliquoted and stored at 4 °C prior to experimental use.

### Animals and experimental design

Juvenile zebrafish (*Danio rerio*) supplied by the Institute of Environment and Ecology, Shandong Normal University (Jinan, China). The fish were maintained in a recirculating aquaculture system equipped with activated carbon filtration and continuous aeration. Water parameters were regulated as follows: temperature 22 ± 2 °C, hardness 250 ± 25 mg/L (as CaCO₃), and pH 7.8 ± 0.2. A photoperiod of 16 h light:8 h dark (lights on at 04:00, off at 20:00) was maintained using overhead lighting (4000 lx intensity at 30 cm above the water surface). Fish were fed twice daily at 09:00 and 16:00, with feeding suspended 24 h prior to experimentation. The experimental design comprised three groups: a phenanthrene (Phe)-exposed group (triplicate tanks), a pyrene (Pyr)-exposed group (triplicate tanks), and a control group (triplicate tanks). Each tank contained five zebrafish (randomly selected; body length 3.5 ± 0.2 cm; approximately 2 months post-hatching). Body weight and volume were measured for all fish prior to exposure. The experiment commenced at 08:00 on Day 1 and continued for 7 days.

The animal experiments in this study have been approved by the Animal Experiment Ethics Review Committee of Shandong Normal University. The approval number is: AEECSDNU2023073.

All methods were carried out in accordance with relevant guidelines and regulations.

All methods are reported in accordance with ARRIVE guidelines (https://arriveguidelines.org).

### Determination of metabolic indicators

**Fig. 1:**
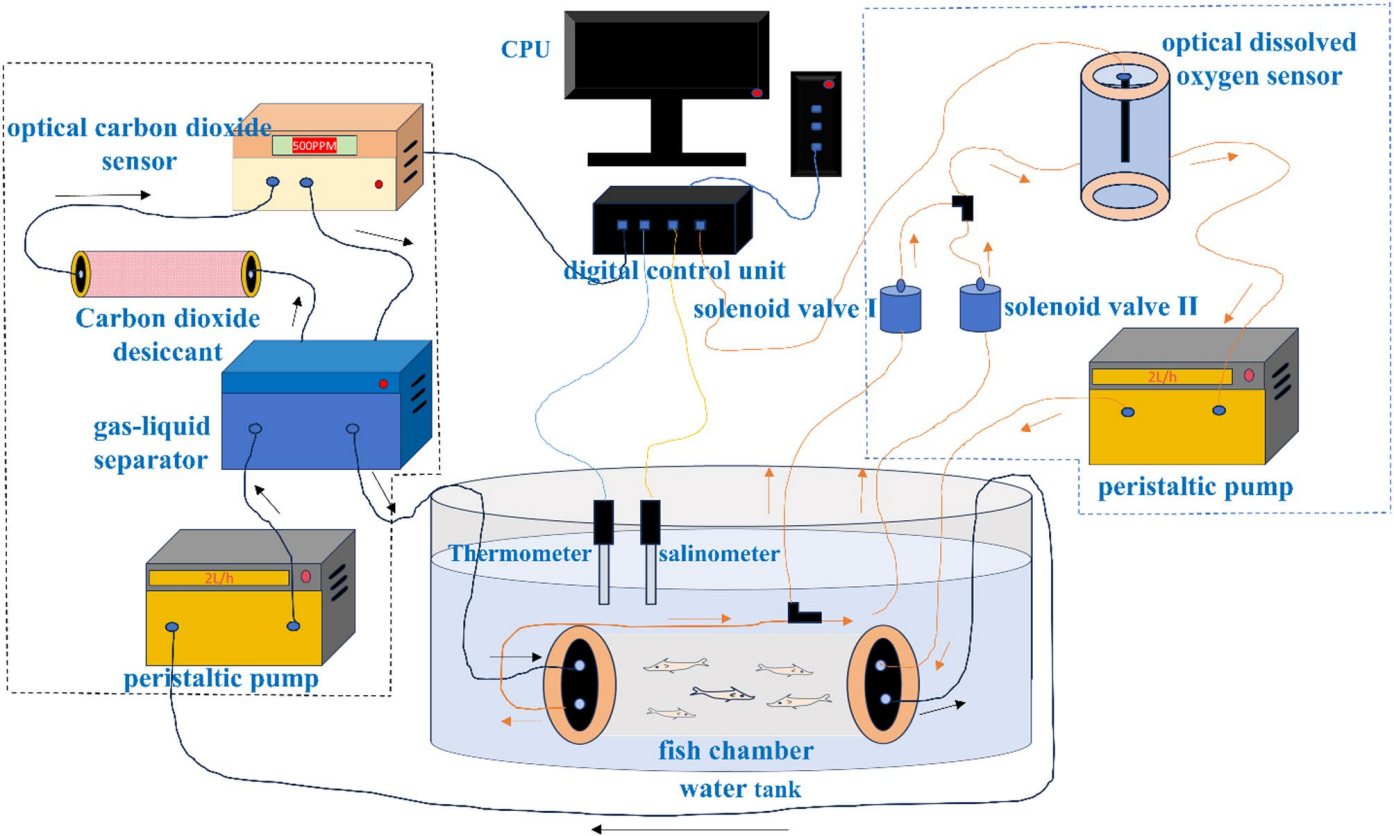
Aquatic metabolism measurement system.

The aquatic metabolic measurement system (Chinese Patent No. 201610344648.X; Fig. 1) developed by the Institute of Environment and Ecology, Shandong Normal University, was employed in this study. This integrated system comprises three subsystems: a data acquisition system, an aquatic oxygen consumption monitoring system, and an aquatic carbon dioxide measurement system. Key components include experimental tanks, fish chambers, optical dissolved oxygen sensors, optical carbon dioxide sensors, peristaltic pumps, solenoid valves, a digital control unit, and a computer. The operational principles of the system are detailed in Supplementary Material 1.

Ammonia-nitrogen excretion rate (AE) in zebrafish was quantified using a multi-parameter water quality analyzer and an ammonia nitrogen assay kit (principles detailed in Supplementary Material 2). Water samples were automatically collected from experimental tanks using a water sampler at 2-h intervals for ammonia-nitrogen analysis. Prior to AE determination, N2 and N3 reagent solutions were prepared following protocols outlined in Supplementary Material 2. For each measurement, 10 mL of ultrapure water and 10 mL of sampled water were aliquoted into separate test tubes. Both tubes were sequentially treated with 1 mL of N3 reagent followed by 1 mL of N2 reagent. After 10 min of incubation and vortex mixing, ammonia-nitrogen concentrations were measured colorimetrically using the multi-parameter analyzer. AE values were subsequently calculated based on these measurements.

### Data analysis

Experimental data were exported to Microsoft Excel for normalization against zebrafish total body volume and mass. Triplicate measurements(There are five fish in a tanks. The average value of each tanks is taken as a repetition). of oxygen concentration (hourly sampling), carbon dioxide concentration (hourly sampling), and ammonia-nitrogen concentration (bi-hourly sampling) underwent statistical analysis to calculate arithmetic means (M) and standard deviations (± SD), representing central tendency and dispersion, respectively. Similar computations were performed for respiratory quotient (hourly sampling) and ammonia quotient (bi-hourly sampling). Temporal variations in these parameters were visualized via real-time line plots using OriginPro 2021 (OriginLab Corporation) for dynamic trend analysis. One-way ANOVA and Mann-Whitney’s test were applied to evaluate differences in OCR, CR, AE, RQ, and AQ between light and dark phases, with statistical significance defined at *p* < 0.05. Circadian rhythmicity in control and treatment groups was further analyzed through autocorrelation analysis and self-organizing map (SOM) neural network implemented in MATLAB R2023a (MathWorks Inc.).

The oxygen consumption rate (OR) is calculated according to Eq. (4):

The rate change of oxygen concentration with time (mg/L/s) is expressed by calculating the average of n slopes (derivatives) with weights (n was set to 3, 5 and 7).

When *n* = 3, the weight is 1

1 $$\:{D}_{i}=\frac{{X}_{i+1}-{X}_{i-1}}{{t}_{i+1}-{t}_{i-1}}$$

When *n* = 5, the weight is 2,1.

2 $$\:{D}_{i}=2\ast\:\frac{{X}_{i+1}-{X}_{i-1}}{{t}_{i+1}-{t}_{i-1}}+\frac{{X}_{i+2}-{X}_{i-2}}{{t}_{i+2}-{t}_{i-2}}$$

When *n* = 7, the weight is 3,2,1.

3 $$\:{D}_{i}=3\ast\:\frac{{X}_{i+1}-{X}_{i-1}}{{t}_{i+1}-{t}_{i-1}}+2\ast\:\frac{{X}_{i+2}-{X}_{i-2}}{{t}_{i+2}-{t}_{i-2}}+\frac{{X}_{i+3}-{X}_{i-3}}{{t}_{i+3}-{t}_{i-3}}$$

t is time (s), x is concentration (mg/L). *Di* is recorded as the average of the n slopes.*Di* is corresponding to the last 300 s of the 450 s time cycle which is to obtain a value every 450 s (dissolved oxygen slope rate of change, mg·L^-1^·s^-1^).

4 $$\:{VO2}_{i}=\frac{\:\text{D}\text{O}\text{S}\text{l}\text{o}\text{p}\text{e}\text{i}\:\times\:\:(\text{R}\text{e}\text{s}\text{p}\text{i}\text{r}\text{V}\text{o}\text{l}\:-\:\text{F}\text{i}\text{s}\text{h}\text{V}\text{o}\text{l})\:\times\:\:3600}{\text{F}\text{i}\text{s}\text{h}\text{W}\text{e}\text{i}\text{g}\text{h}\text{t}}$$

*VO*_*2i*_ is oxygen consumption rate (mg/kg/h), RespirVol is volume of respiratory chamber.

In analogy, the *carbon* dioxide excretion rate (CR) is calculated using Eq. (5):

5 $$\:{VCO2}_{i}=\frac{\:\text{D}\text{C}\text{O}2\text{S}\text{l}\text{o}\text{p}\text{e}\text{i}\:\times\:\:(\text{R}\text{e}\text{s}\text{p}\text{i}\text{r}\text{V}\text{o}\text{l}\:-\:\text{F}\text{i}\text{s}\text{h}\text{V}\text{o}\text{l})\:\times\:\:3600}{\text{F}\text{i}\text{s}\text{h}\text{W}\text{e}\text{i}\text{g}\text{h}\text{t}}$$

The *ammonia* excretion rate (AE) is calculated using Eq. (6):

6 $$\:{VN}_{i}=\frac{\:\text{D}\text{N}\text{S}\text{l}\text{o}\text{p}\text{e}\text{i}\:\times\:\:(\text{R}\text{e}\text{s}\text{p}\text{i}\text{r}\text{V}\text{o}\text{l}\:-\:\text{F}\text{i}\text{s}\text{h}\text{V}\text{o}\text{l})\:\times\:\:3600}{\text{F}\text{i}\text{s}\text{h}\text{W}\text{e}\text{i}\text{g}\text{h}\text{t}}$$

The *respiratory* entropy (RQ) is calculated according to Eq. (7):

7 $$\:RQ=\frac{{VCO}_{2}}{44}/\frac{{VO}_{2}}{32}$$

The ammonia *entropy* (AQ) is calculated according to Eq. (8):

8 $$\:AQ=\frac{VN}{18}/\frac{{VO}_{2}}{32}$$

## Results

### Effects of phe and Pyr exposure on the metabolism of zebrafish

Zebrafish exposed to phenanthrene (Phe, 102 µg/mL) and pyrene (Pyr, 70.6 µg/mL) for 7 days exhibited distinct metabolic alterations (Table 1). Compared to the control group, both Phe- and Pyr-treated groups showed significant suppression in OR (*p* < 0.001) and CR (*p* < 0.05), with OR inhibition markedly stronger than that of CR. Conversely, AE demonstrated a significant increase under Phe and Pyr exposure (*p* < 0.001), indicating a stimulatory effect on nitrogen metabolism. RQ and AQ, as composite indices integrating OR, CR, and AE, were significantly elevated in PAH-treated groups (*p* < 0.001), reflecting coordinated metabolic reprogramming under PAH stress.

**Table 1:** Mean values of OR, CR, AE, RQ and AQ of zebrafish exposed to phe (102 µg/mL) and Pyr (70.6 µg/mL) for 7 consecutive days. ***p < 0.001, *p < 0.05 indicates the difference in mean metabolic values between the treatment and control groups.

	OR (mg/kg/L)	CR (mg/kg/L)	AE (mg/kg/L)	RQ (mg/kg/L)	AQ (mg/kg/L)
Control	1778.57 ± 79.26	127.97 ± 8.50	56.44 ± 3.63	0.056 ± 0.003	0.058 ± 0.002
Phenanthrene (Phe)	444.46 ± 33.05^***^	73.92 ± 10.12^***^	690.60 ± 45.29^***^	0.149 ± 0.010^***^	3.182 ± 0.415^***^
Pyrene (Pyr)	534.26 ± 38.31^***^	107.85 ± 12.71^*^	413.96 ± 36.30^***^	0.192 ± 0.044^***^	1.479 ± 0.217^***^

### Effects of phe and Pyr exposure on OR, CR and AE in zebrafish

**Fig. 2:**
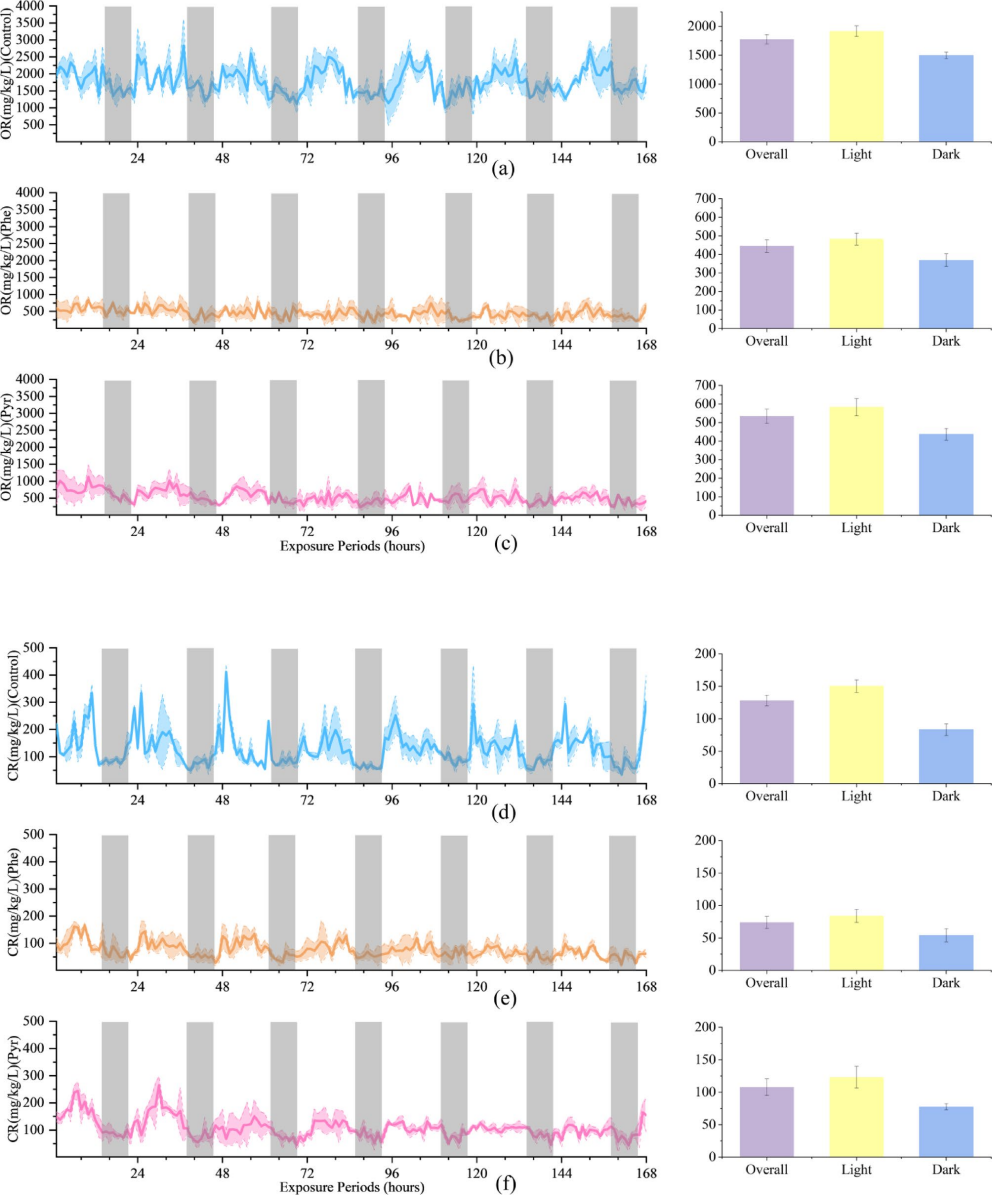

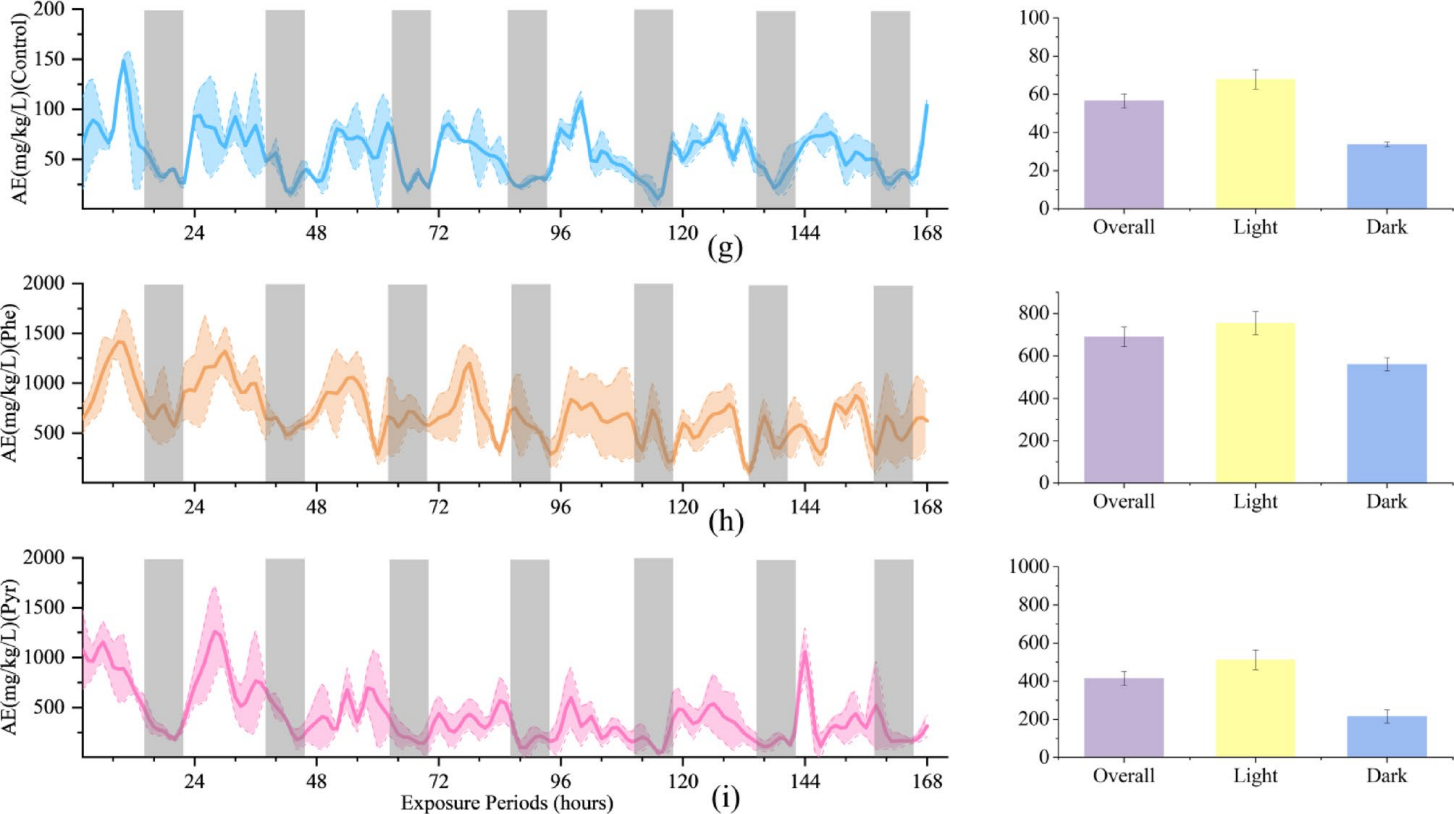
The left panel illustrates the dynamics of OR (**a**, **b**, **c**), CR (**d**, **e**, **f**), and AE (**g**, **h**, **i**) in juvenile zebrafish over the exposure duration, with shaded areas denoting the dark periods during exposure. The right panel presents the Overall, Light, and Dark conditions corresponding to those in the left panel within the exposure time frame.

To elucidate the temporal dynamics of Phe and Pyr effects on zebrafish metabolism, OR, CR, and AE were analyzed across the 7-day exposure period (Fig. 2). Under both PAH treatments, OR values in exposed groups remained significantly lower than those in the control group throughout the experiment. Notably, the Phe-treated group exhibited reduced circadian fluctuation amplitude and stronger inhibitory effects compared to the Pyr-treated group (Fig. 2a, b and c). Similarly, CR levels in treatment groups were consistently suppressed relative to controls. During the initial exposure phase, no significant intergroup differences in CR were observed, but progressive suppression became evident with prolonged exposure, culminating in pronounced inhibition during later stages (Fig. 2d, e and f). The magnitude of CR inhibition was consistently weaker than that of OR, aligning with the comparative effect strengths reported in Table 1. Conversely, AE displayed a distinct temporal pattern. A rapid increase in AE occurred within the first 48 h of PAH exposure, with treated groups maintaining significantly elevated levels compared to controls throughout the experimental duration (Fig. 2g, h and i). Despite PAH-induced alterations in OR, CR, and AE, all three parameters retained robust circadian rhythmicity. These oscillations consistently demonstrated diurnal increases and nocturnal decreases, with peak values predominantly occurring during photophase and troughs aligning with scotophase.

### Effects of phe and Pyr exposure on RQ and AQ in zebrafish

**Fig. 3:**
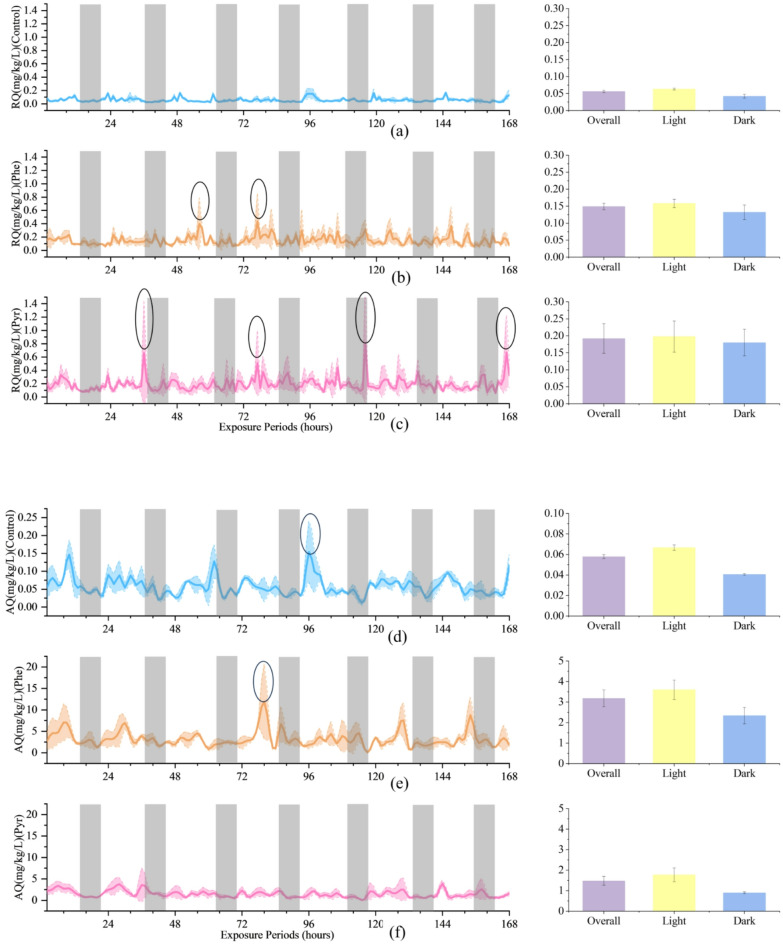
The left panel illustrates the dynamics of RQ (**a**, **b**, **c**), AQ (**d**, **e**, **f**), in zebrafish over the exposure duration, with shaded areas denoting the dark periods during exposure. Black circles indicate where metabolic indicators in zebrafish do not clearly illustrate circadian rhythms.The right panel presents the Overall, Light, and Dark conditions corresponding to those in the left panel within the exposure time frame.

In biological research, RQ and AQ of zebrafish serve as robust composite indices for evaluating physiological status and environmental adaptability. Throughout the experimental period, both RQ and AQ in treated groups significantly exceeded control levels, with Pyr-exposed zebrafish exhibiting higher RQ (Fig. 3a, b and c) and Phe-exposed groups demonstrating elevated AQ (Fig. 3d, e and f). These shifts were mechanistically linked to the marked suppression of OR and CR, coupled with enhanced AE, collectively indicating profound physiological impacts of PAH exposure. Notably, despite these perturbations, RQ and AQ maintained a conserved diurnal rhythm characterized by elevated daytime values and reduced nighttime levels, mirroring control group patterns. Detailed analysis revealed that RQ dynamics paralleled CR variations (supplementary Fig. 1), whereas AQ fluctuations correlated more strongly with AE trends (supplementary Fig. 2).

### Effects of phe and Pyr exposure on the circadian rhythm of zebrafish

**Fig. 4:**
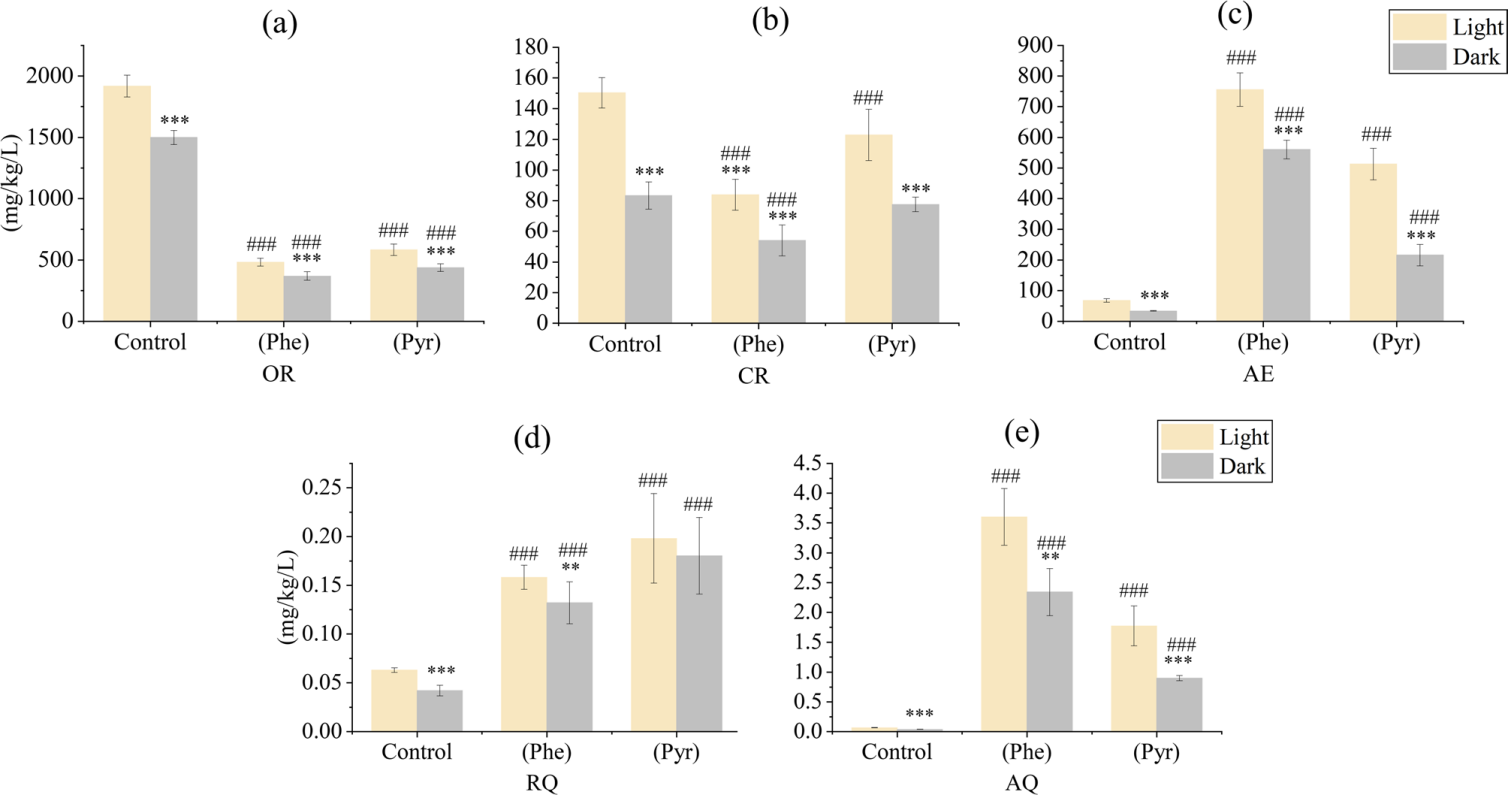
The average of OC(**a**), CR(**b**), AE(**c**), RQ(**d**) and AQ(**e**) of zebrafish with different photoperiod cycles. ^###^*p* < 0.001, indicates the difference in mean metabolic values between the treatment and control groups. *** *p* < 0.001, ** *p* < 0.01 indicates the variability of mean metabolic values for different photoperiods of the same treatment.

To investigate circadian characteristics of metabolic parameters in zebrafish following phenanthrene (Phe) and pyrene (Pyr) exposure, we compared mean values of OR, CR, AE, RQ and AQ under distinct photoperiod conditions (Fig. 4). Results demonstrated significant suppression of OR in both Phe - and Pyr - exposed groups compared to controls (*p* < 0.001; Fig. 4a), with higher OR values observed during photophase than during scotophase (*p* < 0.001; Fig. 4a). CR levels in treated groups were consistently reduced relative to controls across both light phases, yet photophase CR remained significantly elevated over scotophase values (*p* < 0.001; Fig. 4b). AE exhibited marked increases in PAH-treated groups (*p* < 0.001; Fig. 4c) while retaining robust diurnal rhythmicity (*p* < 0.001; Fig. 4c). For RQ and AQ, photophase values in both Phe and Pyr groups exceeded scotophase levels and were significantly elevated compared to controls ( *p* < 0.001; Fig. 4d and e). Pyr exposure induced stronger RQ enhancement, whereas Phe elicited greater AQ stimulation, aligning with trends reported in Fig. 3. Collectively, all metabolic parameters (OR, CR, AE, RQ, AQ) in treated groups displayed higher photophase values than scotophase counterparts. These findings indicate that despite PAH-induced metabolic alterations, zebrafish maintained conserved circadian patterns characterized by elevated daytime and reduced nighttime values.

To elucidate the periodic metabolic changes in zebrafish under phenanthrene (Phe) and pyrene (Pyr) exposure for real-time environmental assessment, autocorrelation analysis and a self-organizing map (SOM) neural network were implemented in MATLAB to investigate circadian rhythmicity of OR, CR, AE, RQ, and AQ.

**Fig. 5:**
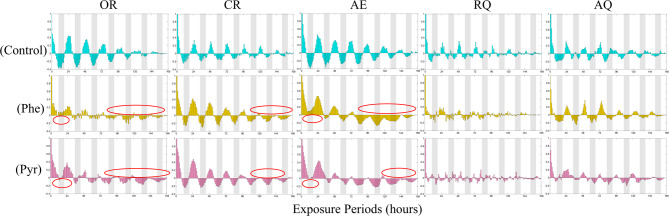
Mean autocorrelation analysis of OC, CR, AE, RQ and AQ in Zebrafish. The red circles represent differences from the control group. Gray shadows represent dark cycles.

Autocorrelation waveform analysis (as shown in Fig. 5) revealed pronounced 24-hour periodicity in OR, CR, AE, RQ, and AQ for both treated and control groups. Control groups exhibited larger amplitude oscillations with distinct regularity across photophase and scotophase, whereas treated groups displayed reduced amplitude, indicating diminished light-dark phase distinctiveness under PAH exposure. Notably, Pyr-treated groups demonstrated autocorrelation profiles closer to controls, with stronger periodicity compared to Phe-treated groups, suggesting relatively preserved circadian rhythmicity. OR, CR, and AE exhibited marked amplitude variations that evolved with exposure duration, while RQ and AQ showed lower-amplitude yet stable oscillations throughout the experiment. Specifically, during early exposure (0–48 h), scotophase OR and AE in treated groups frequently exceeded baseline levels, whereas photophase values fell below baseline in later stages (96–168 h). CR remained comparable to controls during 0–96 h but showed progressive photophase suppression from 120 to 168 h, consistent with trends in Fig. 2b. These findings indicate partial circadian disruption in treated groups, characterized by reduced photophase elevations and anomalous scotophase increases in OR, CR, and AE. Nevertheless, the fundamental photophase-over-scotophase rhythm persisted across all groups. Despite low-amplitude fluctuations, RQ and AQ maintained stable periodicity, attributable to regulatory constraints imposed by coordinated interactions among OR, CR, and AE. This stability was maintained with consistent regularity across the entire observation period. Collectively, both control and treated groups retained circadian rhythms in all metabolic parameters, marked by diurnal peaks and nocturnal troughs. PAH exposure did not significantly disrupt the 24-hour rhythmic patterns of OR, CR, AE, RQ, or AQ in zebrafish.

**Fig. 6:**
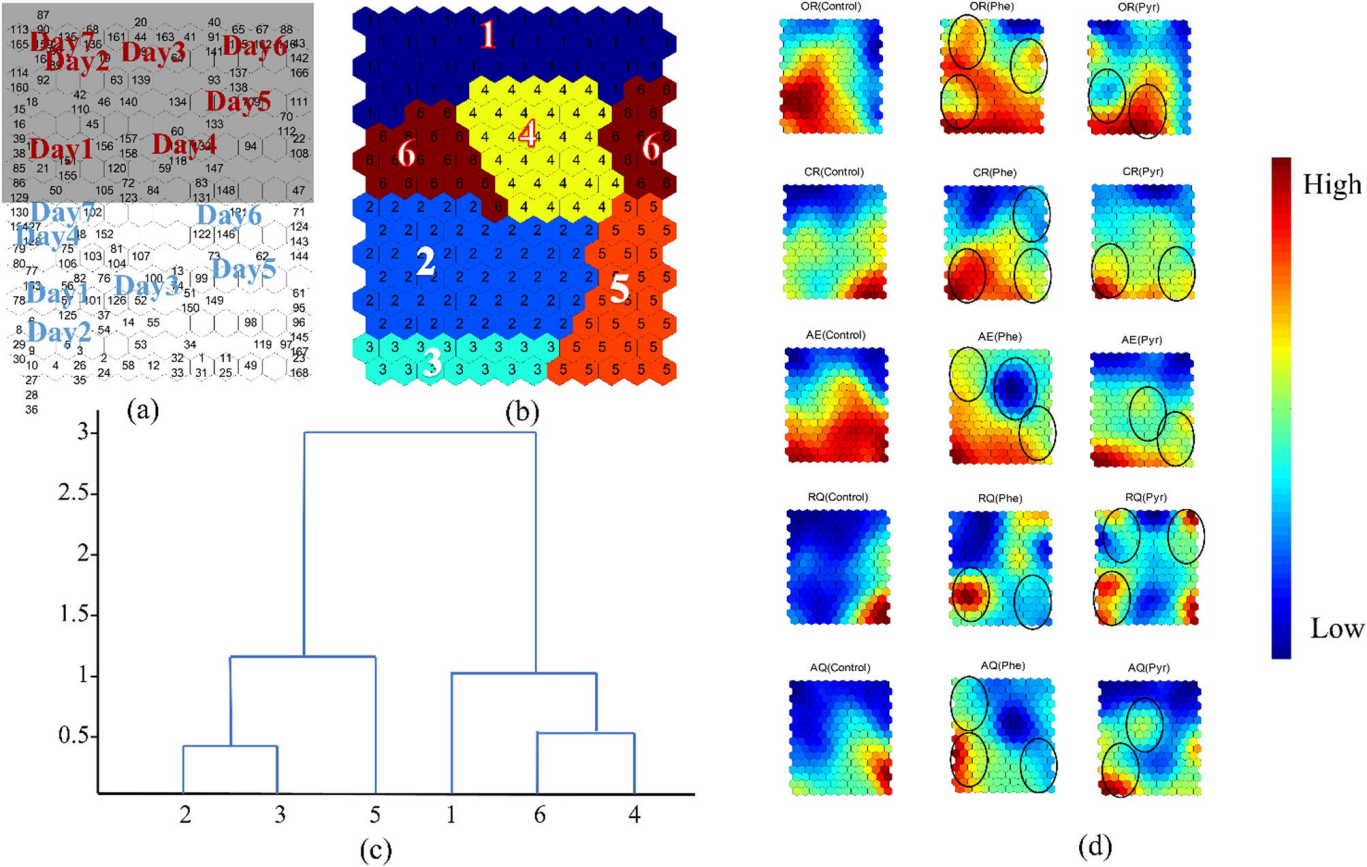
Self-organized mapping (SOM) of OR, CR, AE, RQ, and AQ averages. The black circles indicate areas of difference from the control group.

In the self-organizing map (SOM) neural network analysis, Fig. 6a illustrates temporal distribution across the experimental period, with shaded regions representing scotophase. Figure 6b displays six clusters derived from Ward’s linkage method, where clusters 1, 4, and 6 exhibited high intra-cluster cohesion, as did clusters 2, 3, and 5 (Fig. 6c). Clusters 1, 4, and 6 corresponded to scotophase, while clusters 2, 3, and 5 aligned with photophase. Figure 6d presents SOM topology maps comparing mean values of OR, CR, AE, RQ, and AQ between control and treated groups.

SOM topology analysis revealed similar distribution patterns between PAH-exposed and control groups. Treated groups retained circadian rhythmicity, characterized by lower values in scotophase-associated clusters (1, 4, 6) and elevated levels in photophase-associated clusters (2, 3, 5). Notably, Pyr-treated groups showed closer resemblance to controls in OR, CR, AE, and AQ distributions, whereas Phe-treated groups exhibited greater similarity to controls in RQ patterns. Furthermore, RQ distributions correlated more strongly with CR trends, while AQ aligned with AE dynamics, consistent with the trends in Fig. 3. Overall, short-term Phe and Pyr exposure induced localized perturbations in metabolic rhythms, manifesting as partial photophase suppression and sporadic scotophase elevation, corroborating the findings in Fig. 5. However, the fundamental photophase-elevated and scotophase-depressed circadian pattern remained intact across all groups. These results demonstrate that PAH exposure did not significantly disrupt the circadian stability of OR, CR, AE, RQ, or AQ in zebrafish. This work provides critical insights into metabolic regulation and adaptive responses under environmental PAH stress, advancing mechanistic understanding of aquatic organism resilience to organic pollutants.

## Discussion

This study proposes a real-time biomonitoring approach for assessing early-phase polycyclic aromatic hydrocarbon (PAH) stress in aquatic environments by integrating metabolic parameters (OR, CR, AE, RQ, AQ) and circadian rhythms in zebrafish. Building upon prior work, our team previously identified OR, AE, and AQ as effective biomarkers for evaluating thallium toxicity in zebrafish^48^. Further methodological advancements integrated OR with locomotor responses for real-time cadmium exposure assessment^56^ and combined OR, CR, RQ, and behavioral metrics to monitor copper sulfate contamination^45^. These studies demonstrated pollutant-induced circadian disruptions, consistent with the rhythmic perturbations observed in Figs. 5 and 6 of the current work. Subsequent research utilizing zebrafish respiratory metrics (OR, CR, RQ) and behavior for atrazine monitoring further revealed associations among non-invasive stress biomarkers, oxidative stress indicators, and circadian regulation^57^. In this study, we refined the biomonitoring framework by incorporating circadian rhythmicity and expanded metabolic profiling to investigate PAH exposure. Distinct response patterns in OR, CR, RQ, AE, and AQ were characterized under PAH exposure, affirming the utility of metabolic parameters as indicators of environmental stress. The inclusion of RQ and AQ calculations, although non-significant in this study, provided a more comprehensive perspective on metabolic adaptations to pollutants, while circadian rhythm analysis revealed preserved diurnal oscillations despite acute metabolic perturbations. These findings validate the enhanced sensitivity of integrating real-time metabolic and circadian metrics in real-time biomonitoring technology for aquatic toxicology applications.

Respiratory activity in fish is often the first physiological response to aquatic pollutants, with contaminant exposure potentially altering respiratory rates^58–60^. with oxidative stress mechanisms, as PAHs disrupt redox^61^. To mitigate environmental stress, zebrafish may minimize water influx to modulate respiratory gas exchange, thereby reducing oxygen uptake and maintaining internal homeostasis^62^. This aligns with prior findings demonstrating gill impairment (e.g., gill mucus hypersecretion, epithelial necrosis, and lamellar detachment) in fish following chronic Phe exposure, which directly compromises respiratory efficiency and suppresses OR^63^. Additionally, hypoxia adaptation strategies, such as reduced locomotor activity to lower oxygen demand, may contribute to CR attenuation in contaminated environments. The significant increase in AE under PAH exposure corroborates established toxicological responses, where aquatic organisms enhance ammonia-nitrogen excretion under pollutant stress^64^. Notably, OR, CR, and AE retained circadian rhythmicity during short-term PAH exposure, suggesting latency in pollutant-induced physiological damage. This latency underscores the limited sensitivity of conventional monitoring methods in detecting early-stage sublethal effects^65,66^. Our findings further highlight OR and AE as rapid-response biomarkers during initial exposure (0–48 h), whereas CR demonstrated delayed responsiveness, with peak alterations occurring during later exposure stages. These temporal dynamics position OR and AE as robust indicators for acute contamination alerts, while CR serves as a complementary metric for chronic exposure assessment. The preserved circadian patterns in metabolic parameters, despite PAH-induced perturbations, emphasize the resilience of circadian regulation in aquatic organisms under subacute environmental stress.

RQ, defined as the ratio of carbon dioxide excretion to oxygen consumption over a defined period, serves as an integrative indicator of energy substrate metabolism in zebrafish, reflecting systemic metabolic processes and enabling comprehensive evaluation of physiological states^67^. Similarly, AQ, a composite metric of OR and AE, elucidates the interplay between respiratory metabolism and amino acid deamination^68^. This study observed significant and sustained elevations in both RQ and AQ across the experimental duration under Phe and Pyr exposure, consistent with prior reports of AQ upregulation under environmental stress^69^. Compared to individual metabolic parameters, quotient-based indices (RQ, AQ) provide multidimensional insights into physiological and ecological adaptations, integrating OR, CR, and AE dynamics to precisely assess organismal health and stress resilience. The stability and statistical robustness of RQ and AQ throughout the exposure period further validate their utility as sensitive biomarkers for monitoring PAH contamination. By incorporating OR, CR, AE, RQ, and AQ into biomonitoring frameworks, early detection of PAH contamination becomes feasible, as these metabolic parameters elicit immediate biological responses prior to overt toxic manifestations. Collectively, these metrics position them as effective non-invasive biomarkers for real-time organic pollutant toxicity assessment in aquatic ecosystems.

PAHs pose multifaceted threats to ecosystems and human health, with carcinogenicity being the most prominent risk. This study revealed that 7-day PAH exposure induced partial circadian disruptions in zebrafish metabolic parameters, characterized by suppressed photophase values and elevated scotophase levels. Circadian rhythms, intrinsic biological oscillations governing physiological, behavioral, and metabolic processes, are critical for maintaining organismal homeostasis. Chronic circadian misalignment has been linked to systemic dysfunction and increased susceptibility to malignancies such as breast and colorectal cancers^70–72^. We thus propose that, under the experimental conditions examined, PAHs-induced carcinogenicity may be mechanistically linked to circadian disruption. Further exploration of the interactions between PAHs, circadian disruption, and carcinogenesis could provide valuable insights for both human and environmental health, given that our species is an integral part of the broader ecosystem.Additionally, existing studies have confirmed that PAHs can induce human metabolic disorders through the adverse outcome pathway (AOP), ultimately leading to adverse outcomes such as liver damage, renal necrosis, and cardiac injury^73^. It is noteworthy that metabolic disorder is also one of the characteristics of circadian rhythm disruption. Based on the aforementioned associations, it is hypothesized that circadian rhythm disruption may be another important effect caused by PAHs through AOP. Therefore, in-depth exploration of the mechanism of action between PAHs and AOP is of great significance for assessing the ecological risks of PAH pollution, formulating targeted prevention and control strategies, and protecting biodiversity.

PAHs exhibit extended biological hazard latency, complicating early-stage monitoring during initial exposure. This challenge is intrinsically linked to the pronounced circadian rhythmicity displayed by metabolic indicators in the early phases of PAH exposure. In this study, metabolic responses (OR, CR, AE, RQ, and AQ) were integrated with circadian rhythms to establish a comprehensive response profile. Future studies should validate this methodology through analogous experiments with other non-acute pollutants. Additionally, extending experimental durations could elucidate stage-specific response patterns over exposure durations. Finally, the influence of physicochemical water quality parameters on metabolic and circadian outcomes warrants systematic evaluation.

## Conclusion

This study investigated the metabolic responses of zebrafish to polycyclic aromatic hydrocarbon (PAH) exposure through quantitative analysis of oxygen consumption rate (OR), carbon dioxide excretion rate (CR), ammonia-nitrogen excretion rate (AE), respiratory quotient (RQ), and ammonia quotient (AQ). Key findings revealed that phenanthrene (Phe) and pyrene (Pyr) exposure significantly suppressed OR and CR, with stronger inhibition observed for OR compared to CR. Concurrently, AE exhibited a marked elevation and sustained high levels throughout the exposure period. Both RQ and AQ demonstrated persistent and stable increases, while all parameters retained strong circadian rhythms under PAH exposure.

At present, real-time biomonitoring method based on zebrafish metabolic reactions can effectively reflect the effect of effects of PAH exposure on zebrafish. This helps us detect and deal with organisms in a timely manner before they exhibit adverse reactions in the early stage of pollution. The zebrafish’s OR and AE exhibited significant changes during the early stage of exposure. In contrast, the inhibitory effect of CR was more prominent in the later stage. Consequently, OR and AE can serve as effective biomarkers in the early stage of pollutant exposure, while CR is more appropriate as a monitoring indicator in the later stage of exposure. RQ and AQ under PAHs exposure can fully reflect the respiratory and ammonia metabolism status of zebrafish. Moreover, they are relatively stable. Thus, It is therefore a good indicator of the entire PAH pollution process.

## Supplementary Information

Below is the link to the electronic supplementary material.

Supplementary Material 1

## Data Availability

The datasets used during the current study available from the corresponding author on reasonable request.
