# Supplementary material for: PAHs-induced metabolic aberrations and intact circadian rhythms in zebrafish: a promising approach for aquatic surveillance
Source: Sci Rep. 2025 Aug 19;15:30331. doi: 10.1038/s41598-025-15368-z (PMC12365042; doi:10.1038/s41598-025-15368-z)
Supplement: Supplementary file 1 — Supplementary Material 1 [file 41598_2025_15368_MOESM1_ESM.docx]

**Supplementary Material 1**

**Determination of oxygen and carbon dioxide indicators**

This study utilized the Aquatic Metabolic Measurement System (China Patent No.: 201610344648.X) (Figure 1) developed by the Institute of Environment and Ecology at Shandong Normal University. The system consists of three integrated components: a data acquisition module, an aquatic oxygen consumption measurement module, and an aquatic carbon dioxide measurement module.

The aquatic O_2_ consumption measurement module employs an optical dissolved oxygen sensor as its core component. Based on the fluorescence quenching principle of specific dye substances in physics, blue light emitted from a light-emitting diode irradiates the reactive fluorescent material on the inner surface of the sensor probe, inducing energy level transitions and subsequent redlight emission. The phase shift between the excitation (blue) and emission (red) wavelengths exhibits a direct linear correlation with dissolved oxygen concentration in water, enabling precise quantification of oxygen molecules. The aquatic carbon dioxide measurement module features an optical CO₂ sensor utilizing a single-beam dual-wavelength non-dispersive infrared spectroscopy technique for real-time monitoring. Given CO₂'s strong absorption at 4.26 nm infrared wavelength, the sensor incorporates an internal light source, wavelength-specific filter, and detector. Post gas-liquid separation, gaseous samples are channeled into the analyzer's measurement chamber where the filter isolates 4.26 nm radiation. The detector measures transmitted light intensity, which is converted into proportional electrical signals for CO₂ concentration determination. Analog signals from all sensors are digitized by a digital control unit and stored in a computer system (Fig.1).

Aquatic Metabolic Measurement System Configuration: The fish chamber is submerged within a water tank, sealed by a dual-ported lid connecting to the Aquatic CO₂ Measurement System (left port) and Aquatic O_2_ Consumption Measurement System (right port). Following signal conversion by the data control unit, oxygen and carbon dioxide concentration values are retrieved and displayed on the computer interface.

Aquatic CO₂ Measurement System: Water from the fish chamber is pumped via a rubber tube (connected through the lower-right port) to a gas-liquid separator using a peristaltic pump. The evolved gaseous CO₂ is dried by a CO₂ desiccant before entering the CO₂ analyzer, which displays real-time concentration in ppm. The degassed water then recirculates to the upper-left port of the fish chamber through the gas-liquid separator.

Aquatic O_2_ Consumption Measurement System: Water from the fish chamber flows through a rubber tube (connected via the lower-left port) to a dissolved O_2_ sensor controlled by a solenoid valve, then through a peristaltic pump, and back to the upper-right port of the fish chamber.

This system employs two solenoid valves to regulate flow modes: Solenoid Valve I, activates closed-loop circulation mode, maintaining a sealed monitoring system for 300 seconds. Solenoid Valve II, engages open-flow flushing mode, directing fresh water from the tank through the sensor for 150 seconds before returning it to the tank.


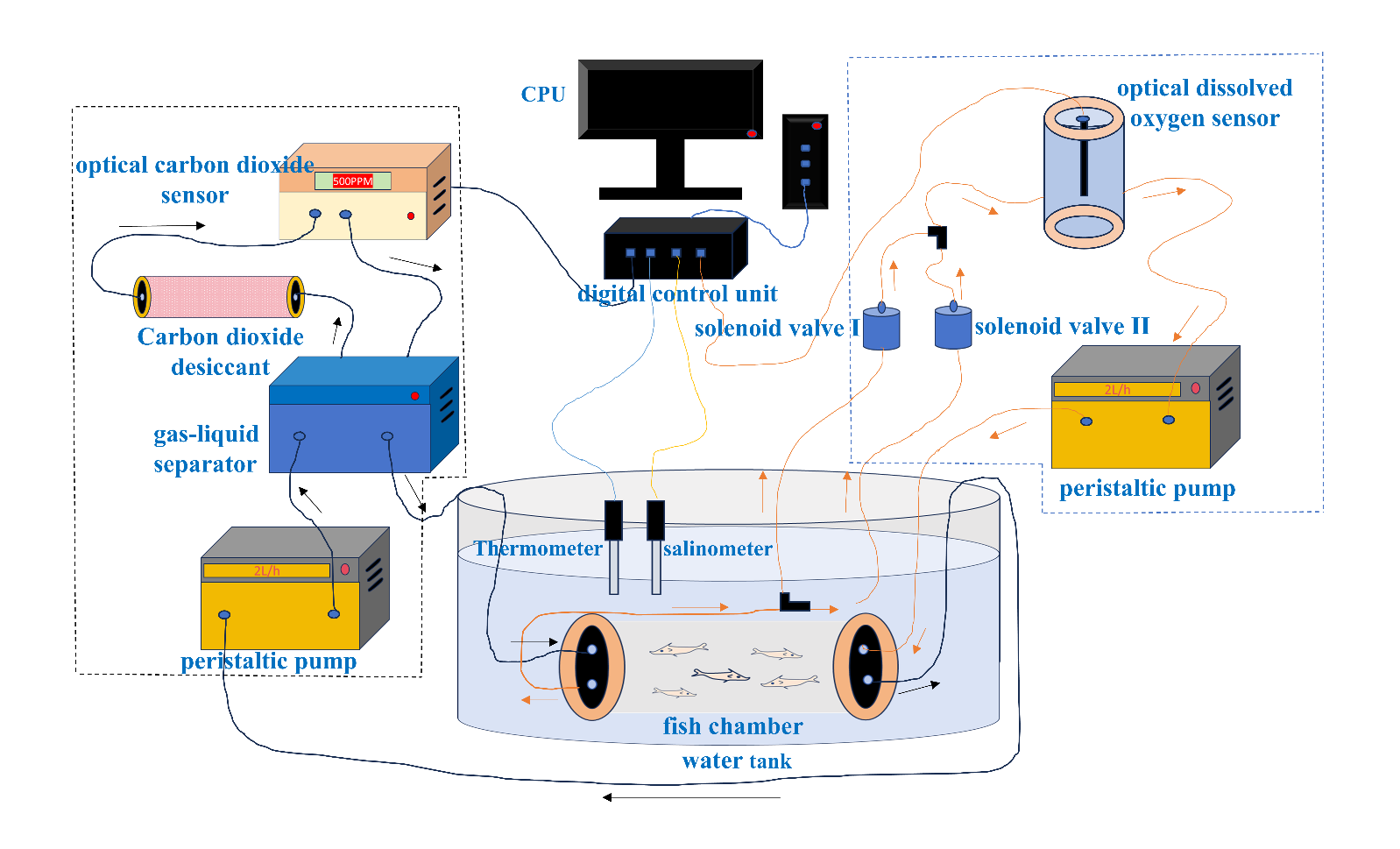


Fig.1. Aquatic Metabolic Measurement System

**Supplementary Material 2**

**Determination of ammonia nitrogen index**

We use a Multi-Parameter Water Quality Analyzer to determine the content of ammonia nitrogen in water. The main principle is: the alkaline solution of mercury iodide and potassium iodide reacts with ammonia to produce a light reddish-brown colloid compound, and its chroma is proportional to the content of ammonia nitrogen. The multi-water quality detector can measure its absorbance in the range of wave 410~425nm, and calculate its content according to the absorbance. The instrument has the characteristics of high precision, simple operation and sensitive instrument, which can be applied to the detection of surface water, groundwater, industrial waste water and domestic sewage.

The preparation method of LH-N2N3-100 ammonia nitrogen kit is as follows: Pour the whole bottle of LH-N2-100 powder into a beaker, measure 100mL of ammonia-free water, pour a small amount (about 30mL) of ammonia-free water first, stir and dissolve fully, then cool, then pour the remaining ammonia-free water, stir well, the solution is yellow and transparent, and let it stand overnight before use; Pour the whole bottle of LH-N3-100 powder into the beaker, measure 100mL of ammonia-free water into the beaker, stir and dissolve fully, the solution is colorless and transparent.

**Supplementary Material 3**

1. **Comparison of CR and RQ**


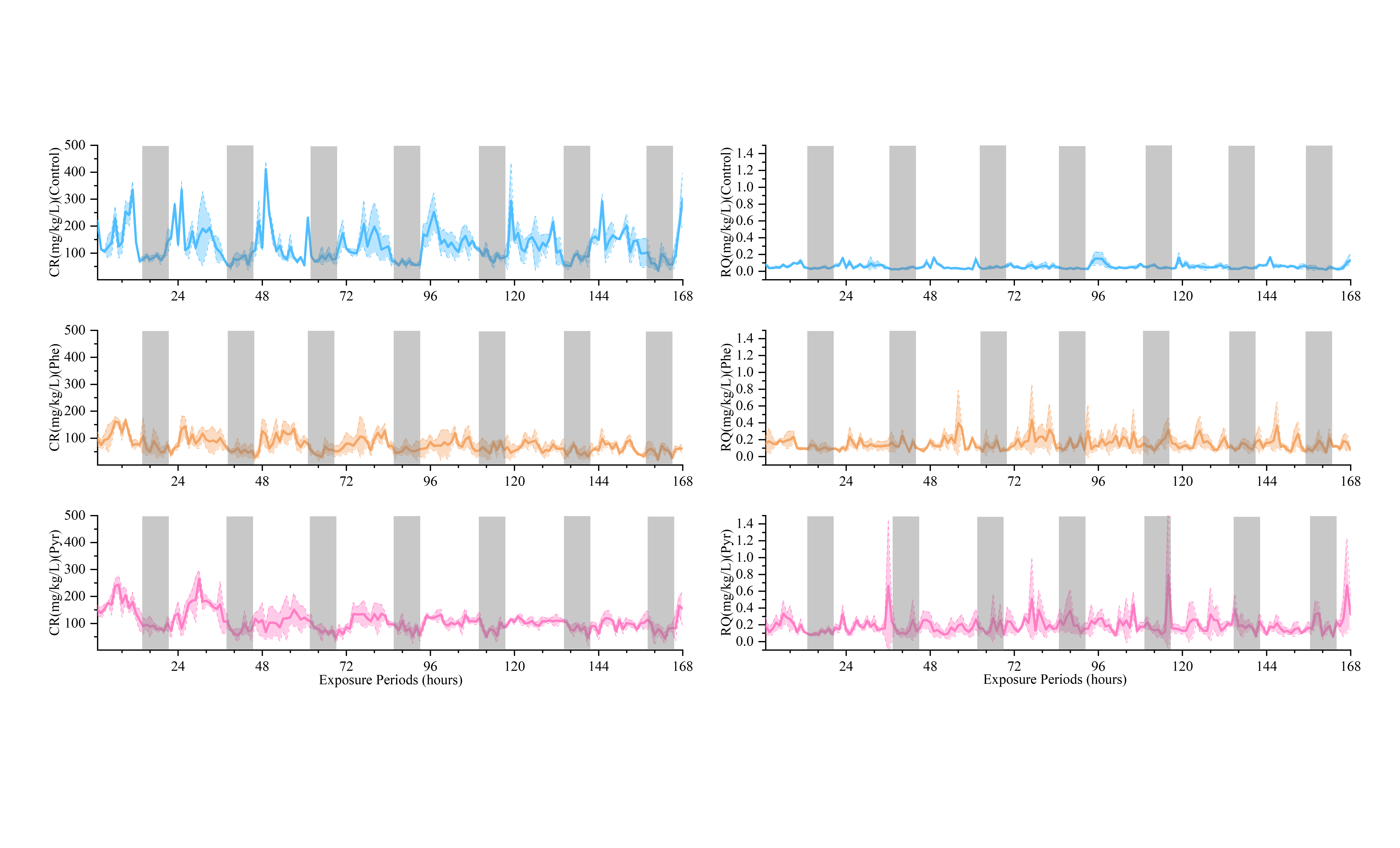


**supplementary fig. 1.** Temporal dynamics of CR and RQ of zebrafish with exposure time. Shaded areas indicate periods of darkness during exposure.

1. **Contrast chart of AE and AQ**


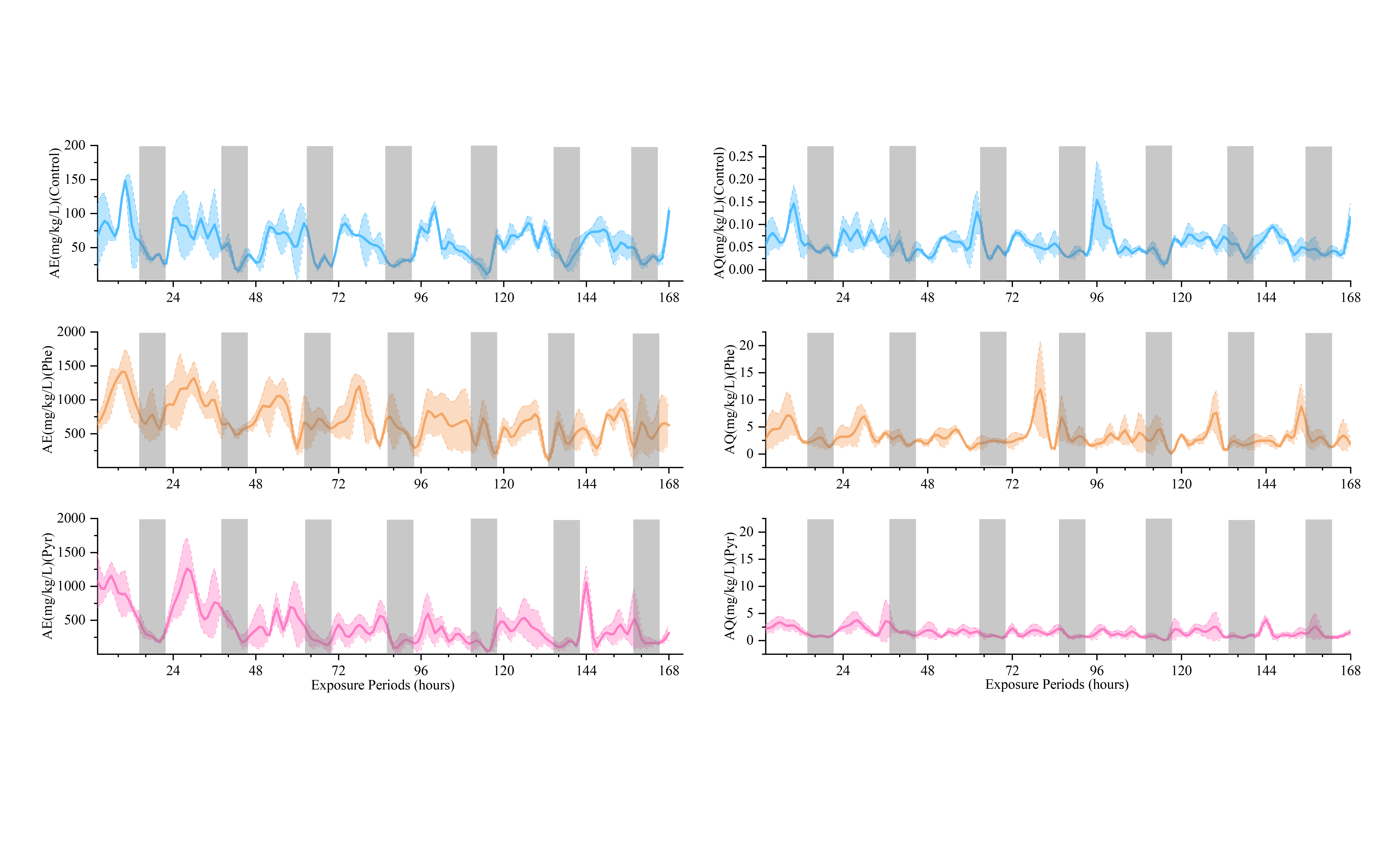


**supplementary fig. 1.** Temporal dynamics of AE and AQ of zebrafish with exposure time. Shaded areas indicate periods of darkness during exposure.
